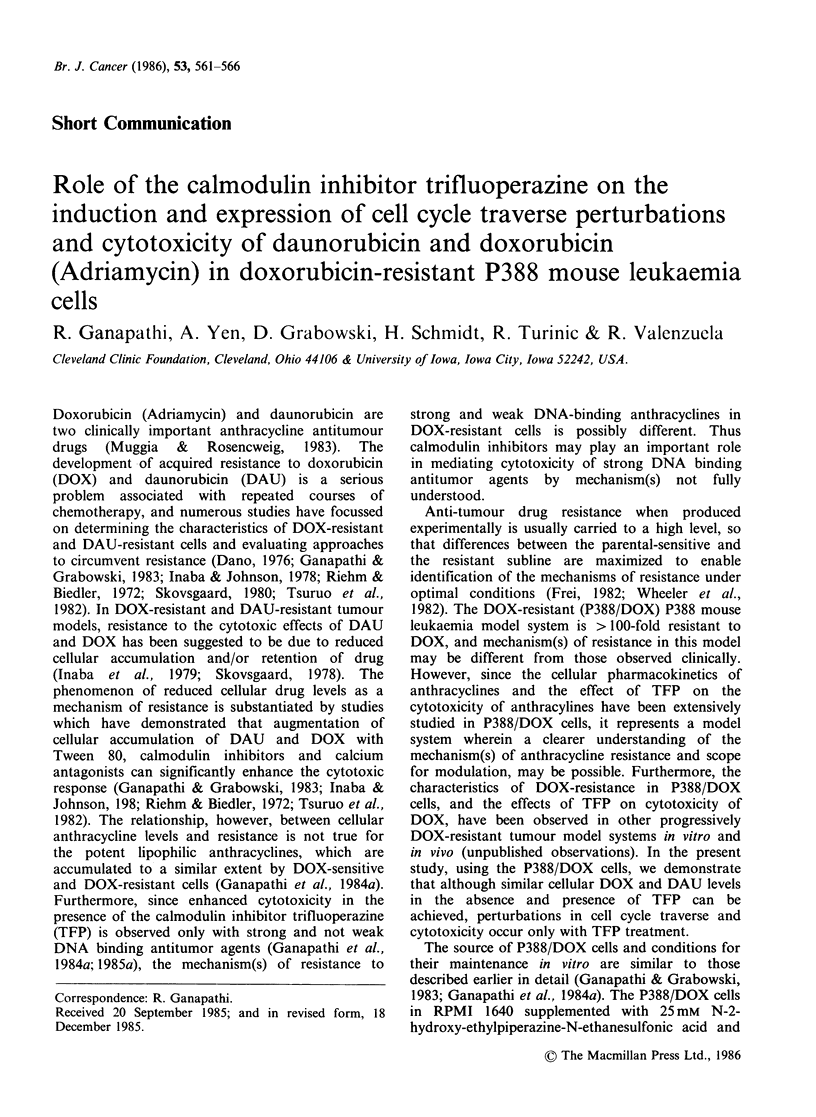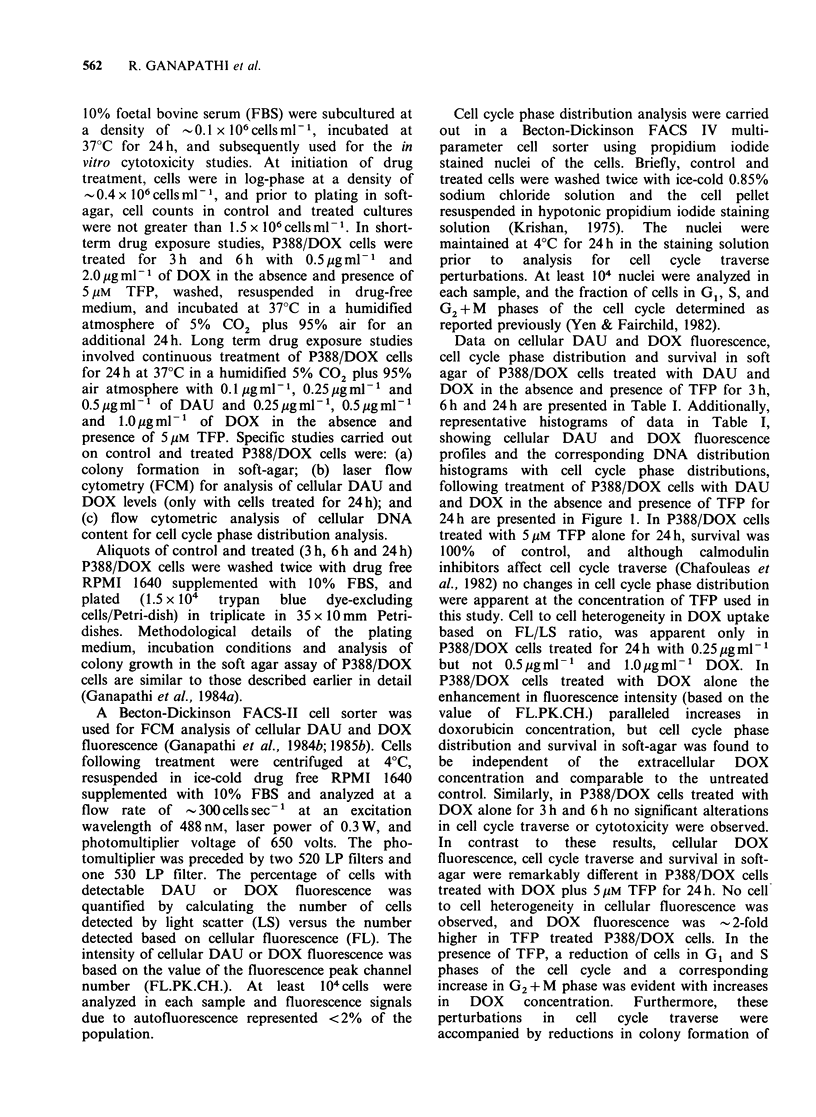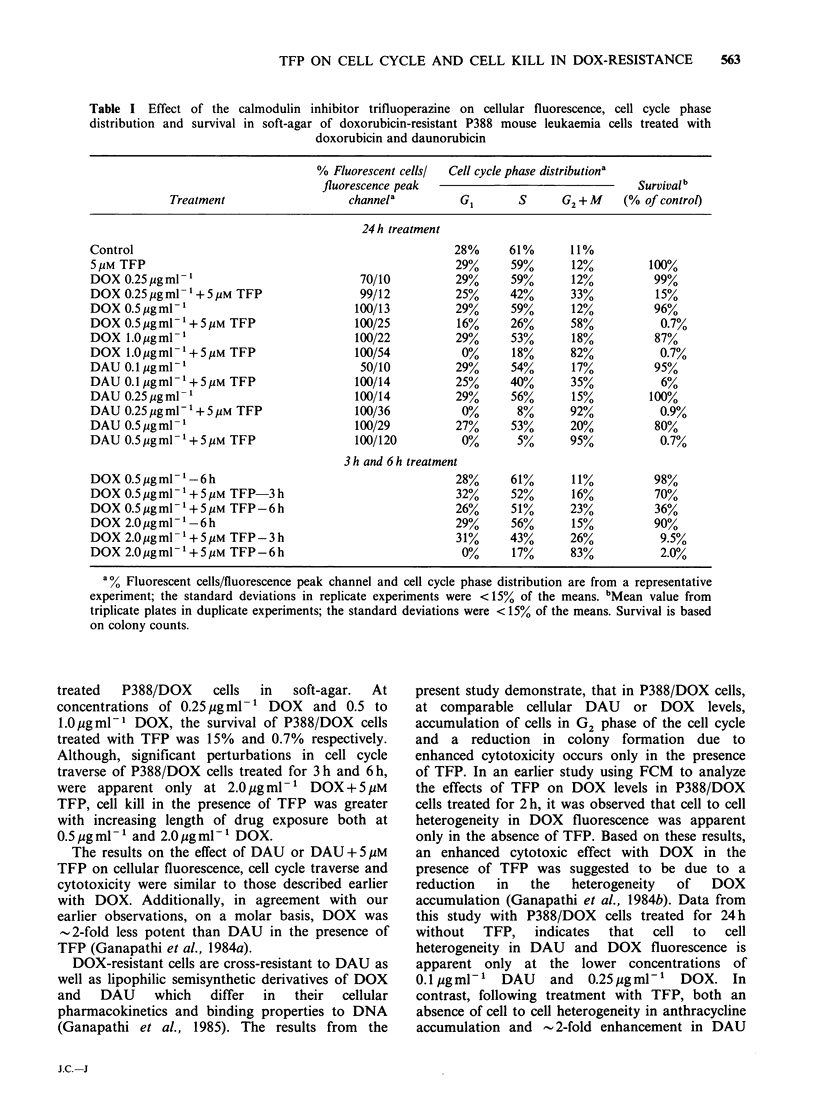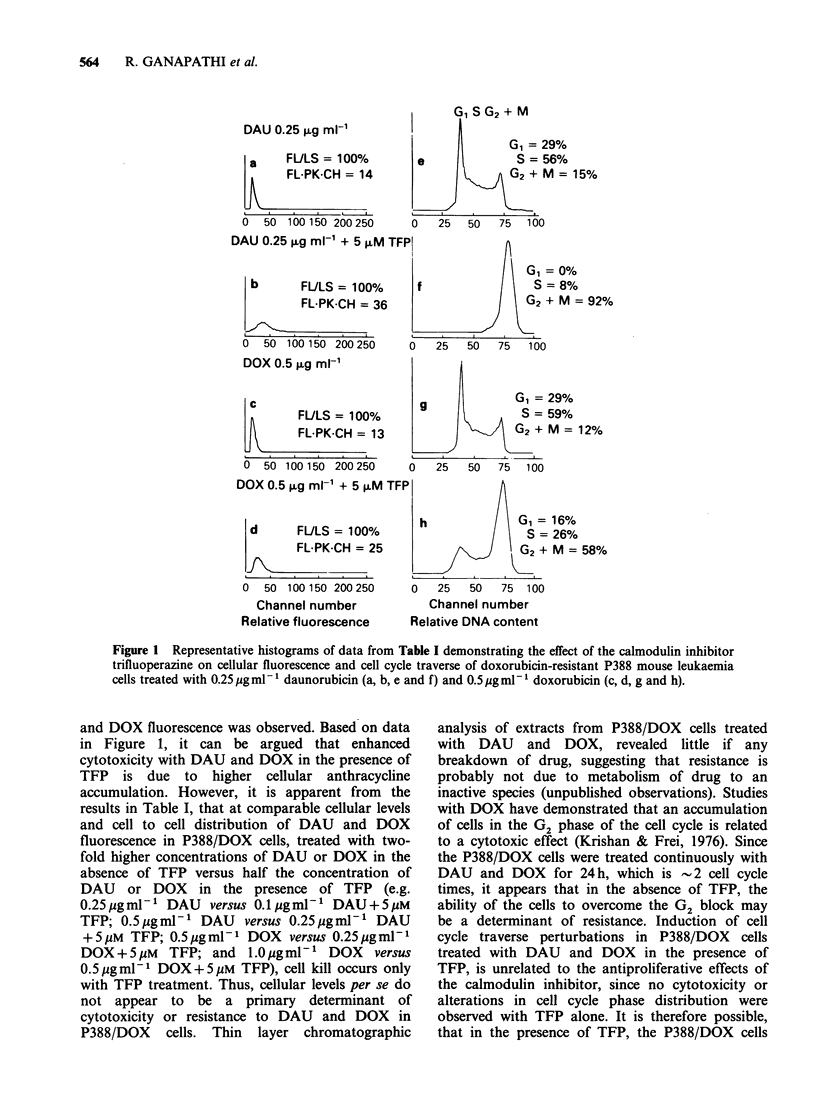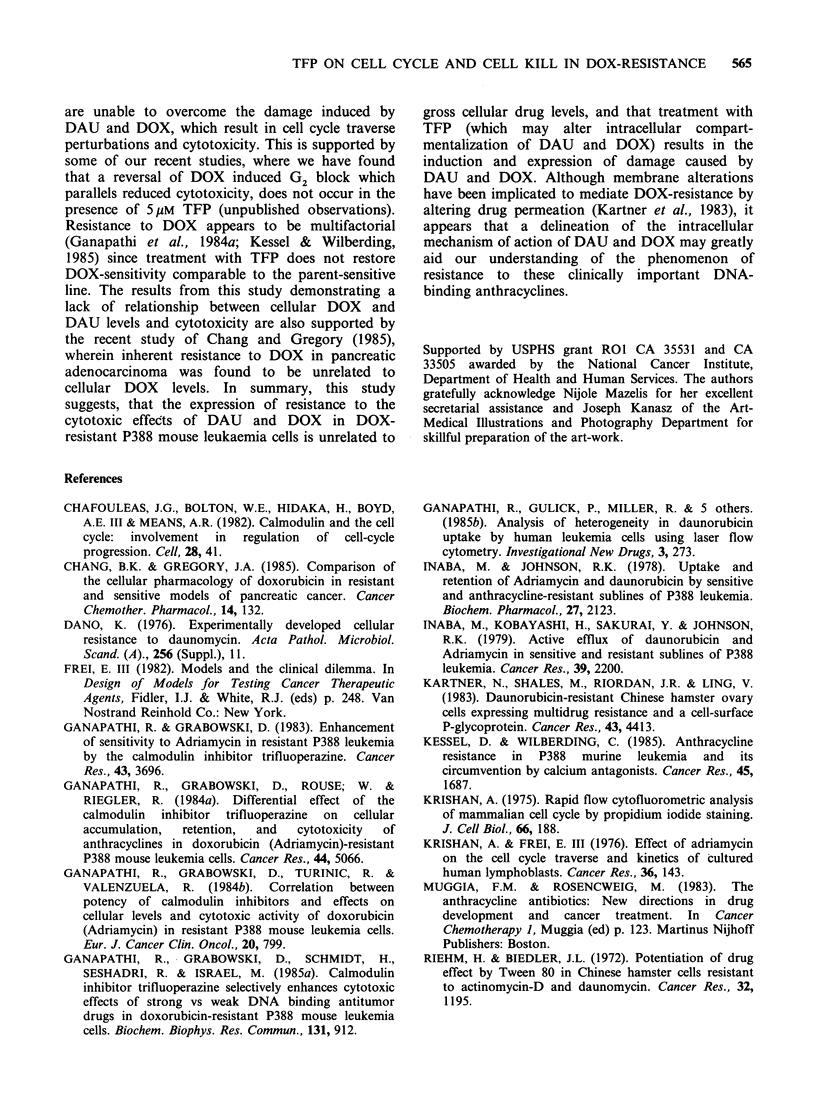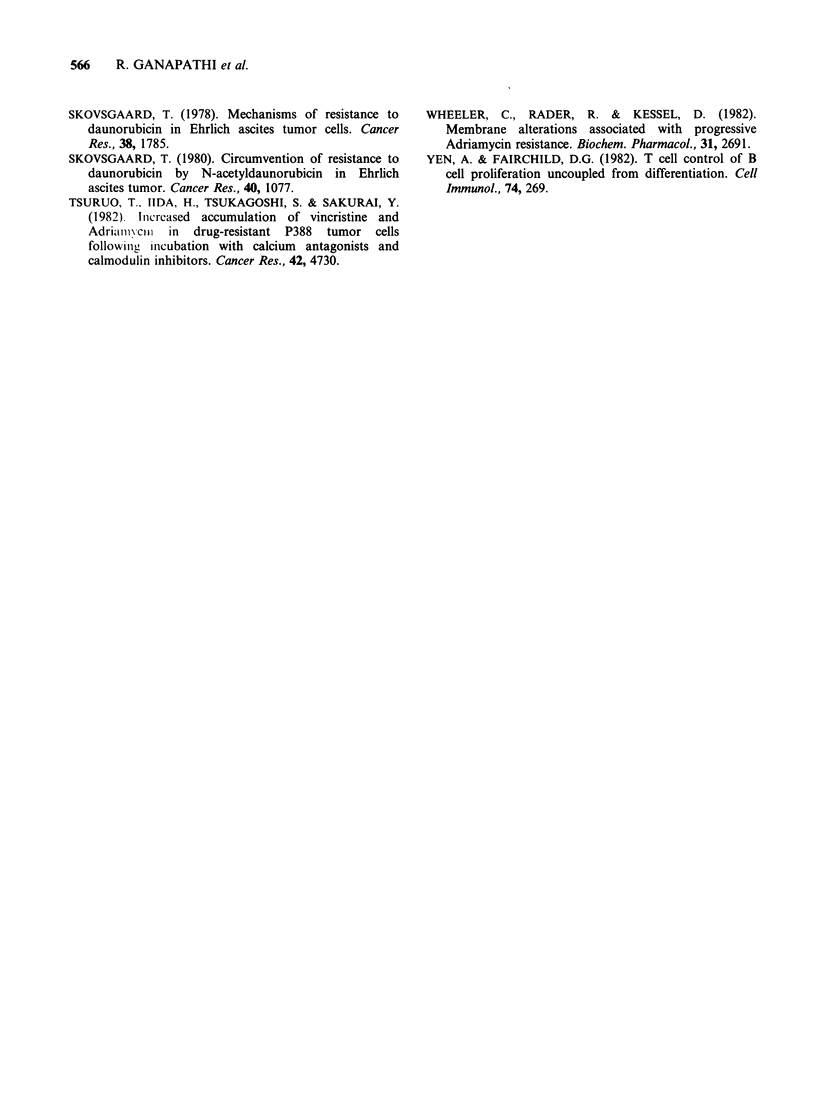# Role of the calmodulin inhibitor trifluoperazine on the induction and expression of cell cycle traverse perturbations and cytotoxicity of daunorubicin and doxorubicin (adriamycin) in doxorubicin-resistant P388 mouse leukaemia cells.

**DOI:** 10.1038/bjc.1986.88

**Published:** 1986-04

**Authors:** R. Ganapathi, A. Yen, D. Grabowski, H. Schmidt, R. Turinic, R. Valenzuela


*Br. J. Cancer* (1986), **53**, 561–566

### Short Communication

### Role of the calmodulin inhibitor trifluoperazine on the induction and expression of cell cycle traverse perturbations and cytotoxicity of daunorubicin and doxorubicin (Adriamycin) in doxorubicin-resistant P388 mouse leukaemia cells

R. Ganapathi, A. Yen, D. Grabowski, H. Schmidt, R. Turinic & R. Valenzuela

*Cleveland Clinic Foundation, Cleveland, Ohio 44106 & University of Iowa, Iowa City, Iowa 52242, USA.*

Doxorubicin (Adriamycin) and daunorubicin are two clinically important anthracycline antitumour drugs (Muggia & Rosencweig, 1983). The development of acquired resistance to doxorubicin (DOX) and daunorubicin (DAU) is a serious problem associated with repeated courses of chemotherapy, and numerous studies have focussed on determining the characteristics of DOX-resistant and DAU-resistant cells and evaluating approaches to circumvent resistance (Dano, 1976; Ganapathi & Grabowski, 1983; Inaba & Johnson, 1978; Riehm & Biedler, 1972; Skovsgaard, 1980; Tsuruo *et al.*, 1982). In DOX-resistant and DAU-resistant tumour models, resistance to the cytotoxic effects of DAU and DOX has been suggested to be due to reduced cellular accumulation and/or retention of drug (Inaba *et al.*, 1979; Skovsgaard, 1978). The phenomenon of reduced cellular drug levels as a mechanism of resistance is substantiated by studies which have demonstrated that augmentation of cellular accumulation of DAU and DOX with Tween 80, calmodulin inhibitors and calcium antagonists can significantly enhance the cytotoxic response (Ganapathi & Grabowski, 1983; Inaba & Johnson, 198; Riehm & Biedler, 1972; Tsuruo *et al.*, 1982). The relationship, however, between cellular anthracycline levels and resistance is not true for the potent lipophilic anthracyclines, which are accumulated to a similar extent by DOX-sensitive and DOX-resistant cells (Ganapathi *et al.*, 1984*a*). Furthermore, since enhanced cytotoxicity in the presence of the calmodulin inhibitor trifluoperazine (TFP) is observed only with strong and not weak DNA binding antitumor agents (Ganapathi *et al.*, 1984*a*; 1985*a*), the mechanism(s) of resistance to strong and weak DNA-binding anthracyclines in DOX-resistant cells is possibly different. Thus calmodulin inhibitors may play an important role in mediating cytotoxicity of strong DNA binding antitumor agents by mechanism(s) not fully understood.

Anti-tumour drug resistance when produced experimentally is usually carried to a high level, so that differences between the parental-sensitive and the resistant subline are maximized to enable identification of the mechanisms of resistance under optimal conditions (Frei, 1982; Wheeler *et al.*, 1982). The DOX-resistant (P388/DOX) P388 mouse leukaemia model system is >100-fold resistant to DOX, and mechanism(s) of resistance in this model may be different from those observed clinically. However, since the cellular pharmacokinetics of anthracyclines and the effect of TFP on the cytotoxicity of anthracylines have been extensively studied in P388/DOX cells, it represents a model system wherein a clearer understanding of the mechanism(s) of anthracycline resistance and scope for modulation, may be possible. Furthermore, the characteristics of DOX-resistance in P388/DOX cells, and the effects of TFP on cytotoxicity of DOX, have been observed in other progressively DOX-resistant tumour model systems *in vitro* and *in vivo* (unpublished observations). In the present study, using the P388/DOX cells, we demonstrate that although similar cellular DOX and DAU levels in the absence and presence of TFP can be achieved, perturbations in cell cycle traverse and cytotoxicity occur only with TFP treatment.

The source of P388/DOX cells and conditions for their maintenance *in vitro* are similar to those described earlier in detail (Ganapathi & Grabowski, 1983; Ganapathi *et al.*, 1984*a*). The P388/DOX cells in RPMI 1640 supplemented with 25 mM N-2-hydroxy-ethylpiperazine-N-ethanesulfonic acid and

Correspondence: R. Ganapathi.
Received 20 September 1985; and in revised form, 18 December 1985.

© The Macmillan Press Ltd., 1986

10% foetal bovine serum (FBS) were subcultured at a density of $\sim 0.1 \times 10^6$ cells ml$^{-1}$, incubated at 37°C for 24 h, and subsequently used for the *in vitro* cytotoxicity studies. At initiation of drug treatment, cells were in log-phase at a density of $\sim 0.4 \times 10^6$ cells ml$^{-1}$, and prior to plating in soft-agar, cell counts in control and treated cultures were not greater than $1.5 \times 10^6$ cells ml$^{-1}$. In short-term drug exposure studies, P388/DOX cells were treated for 3 h and 6 h with 0.5 $\mu$g ml$^{-1}$ and 2.0 $\mu$g ml$^{-1}$ of DOX in the absence and presence of 5 $\mu$M TFP, washed, resuspended in drug-free medium, and incubated at 37°C in a humidified atmosphere of 5% $CO_2$ plus 95% air for an additional 24 h. Long term drug exposure studies involved continuous treatment of P388/DOX cells for 24 h at 37°C in a humidified 5% $CO_2$ plus 95% air atmosphere with 0.1 $\mu$g ml$^{-1}$, 0.25 $\mu$g ml$^{-1}$ and 0.5 $\mu$g ml$^{-1}$ of DAU and 0.25 $\mu$g ml$^{-1}$, 0.5 $\mu$g ml$^{-1}$ and 1.0 $\mu$g ml$^{-1}$ of DOX in the absence and presence of 5 $\mu$M TFP. Specific studies carried out on control and treated P388/DOX cells were: (a) colony formation in soft-agar; (b) laser flow cytometry (FCM) for analysis of cellular DAU and DOX levels (only with cells treated for 24 h); and (c) flow cytometric analysis of cellular DNA content for cell cycle phase distribution analysis.

Aliquots of control and treated (3 h, 6 h and 24 h) P388/DOX cells were washed twice with drug free RPMI 1640 supplemented with 10% FBS, and plated ($1.5 \times 10^4$ trypan blue dye-excluding cells/Petri-dish) in triplicate in 35 × 10 mm Petri-dishes. Methodological details of the plating medium, incubation conditions and analysis of colony growth in the soft agar assay of P388/DOX cells are similar to those described earlier in detail (Ganapathi *et al.*, 1984*a*).

A Becton-Dickinson FACS-II cell sorter was used for FCM analysis of cellular DAU and DOX fluorescence (Ganapathi *et al.*, 1984*b*; 1985*b*). Cells following treatment were centrifuged at 4°C, resuspended in ice-cold drug free RPMI 1640 supplemented with 10% FBS and analyzed at a flow rate of $\sim 300$ cells sec$^{-1}$ at an excitation wavelength of 488 nM, laser power of 0.3 W, and photomultiplier voltage of 650 volts. The photomultiplier was preceded by two 520 LP filters and one 530 LP filter. The percentage of cells with detectable DAU or DOX fluorescence was quantified by calculating the number of cells detected by light scatter (LS) versus the number detected based on cellular fluorescence (FL). The intensity of cellular DAU or DOX fluorescence was based on the value of the fluorescence peak channel number (FL.PK.CH.). At least $10^4$ cells were analyzed in each sample and fluorescence signals due to autofluorescence represented <2% of the population.

Cell cycle phase distribution analysis were carried out in a Becton-Dickinson FACS IV multi-parameter cell sorter using propidium iodide stained nuclei of the cells. Briefly, control and treated cells were washed twice with ice-cold 0.85% sodium chloride solution and the cell pellet resuspended in hypotonic propidium iodide staining solution (Krishan, 1975). The nuclei were maintained at 4°C for 24 h in the staining solution prior to analysis for cell cycle traverse perturbations. At least $10^4$ nuclei were analyzed in each sample, and the fraction of cells in $G_1$, S, and $G_2+M$ phases of the cell cycle determined as reported previously (Yen & Fairchild, 1982).

Data on cellular DAU and DOX fluorescence, cell cycle phase distribution and survival in soft agar of P388/DOX cells treated with DAU and DOX in the absence and presence of TFP for 3 h, 6 h and 24 h are presented in Table I. Additionally, representative histograms of data in Table I, showing cellular DAU and DOX fluorescence profiles and the corresponding DNA distribution histograms with cell cycle phase distributions, following treatment of P388/DOX cells with DAU and DOX in the absence and presence of TFP for 24 h are presented in Figure 1. In P388/DOX cells treated with 5 $\mu$M TFP alone for 24 h, survival was 100% of control, and although calmodulin inhibitors affect cell cycle traverse (Chafouleas *et al.*, 1982) no changes in cell cycle phase distribution were apparent at the concentration of TFP used in this study. Cell to cell heterogeneity in DOX uptake based on FL/LS ratio, was apparent only in P388/DOX cells treated for 24 h with 0.25 $\mu$g ml$^{-1}$ but not 0.5 $\mu$g ml$^{-1}$ and 1.0 $\mu$g ml$^{-1}$ DOX. In P388/DOX cells treated with DOX alone the enhancement in fluorescence intensity (based on the value of FL.PK.CH.) paralleled increases in doxorubicin concentration, but cell cycle phase distribution and survival in soft-agar was found to be independent of the extracellular DOX concentration and comparable to the untreated control. Similarly, in P388/DOX cells treated with DOX alone for 3 h and 6 h no significant alterations in cell cycle traverse or cytotoxicity were observed. In contrast to these results, cellular DOX fluorescence, cell cycle traverse and survival in soft-agar were remarkably different in P388/DOX cells treated with DOX plus 5 $\mu$M TFP for 24 h. No cell to cell heterogeneity in cellular fluorescence was observed, and DOX fluorescence was $\sim$2-fold higher in TFP treated P388/DOX cells. In the presence of TFP, a reduction of cells in $G_1$ and S phases of the cell cycle and a corresponding increase in $G_2+M$ phase was evident with increases in DOX concentration. Furthermore, these perturbations in cell cycle traverse were accompanied by reductions in colony formation of

**Table I** Effect of the calmodulin inhibitor trifluoperazine on cellular fluorescence, cell cycle phase distribution and survival in soft-agar of doxorubicin-resistant P388 mouse leukaemia cells treated with doxorubicin and daunorubicin

| *Treatment* | *% Fluorescent cells/ fluorescence peak channel*[a] | *Cell cycle phase distribution*[a] $G_1$ | $S$ | $G_2+M$ | *Survival*[b] *(% of control)* |
|---|---|---|---|---|---|
| | *24 h treatment* | | | | |
| Control | | 28% | 61% | 11% | |
| 5 μM TFP | | 29% | 59% | 12% | 100% |
| DOX 0.25 μg ml$^{-1}$ | 70/10 | 29% | 59% | 12% | 99% |
| DOX 0.25 μg ml$^{-1}$ + 5 μM TFP | 99/12 | 25% | 42% | 33% | 15% |
| DOX 0.5 μg ml$^{-1}$ | 100/13 | 29% | 59% | 12% | 96% |
| DOX 0.5 μg ml$^{-1}$ + 5 μM TFP | 100/25 | 16% | 26% | 58% | 0.7% |
| DOX 1.0 μg ml$^{-1}$ | 100/22 | 29% | 53% | 18% | 87% |
| DOX 1.0 μg ml$^{-1}$ + 5 μM TFP | 100/54 | 0% | 18% | 82% | 0.7% |
| DAU 0.1 μg ml$^{-1}$ | 50/10 | 29% | 54% | 17% | 95% |
| DAU 0.1 μg ml$^{-1}$ + 5 μM TFP | 100/14 | 25% | 40% | 35% | 6% |
| DAU 0.25 μg ml$^{-1}$ | 100/14 | 29% | 56% | 15% | 100% |
| DAU 0.25 μg ml$^{-1}$ + 5 μM TFP | 100/36 | 0% | 8% | 92% | 0.9% |
| DAU 0.5 μg ml$^{-1}$ | 100/29 | 27% | 53% | 20% | 80% |
| DAU 0.5 μg ml$^{-1}$ + 5 μM TFP | 100/120 | 0% | 5% | 95% | 0.7% |
| | *3 h and 6 h treatment* | | | | |
| DOX 0.5 μg ml$^{-1}$ – 6 h | | 28% | 61% | 11% | 98% |
| DOX 0.5 μg ml$^{-1}$ + 5 μM TFP—3 h | | 32% | 52% | 16% | 70% |
| DOX 0.5 μg ml$^{-1}$ + 5 μM TFP – 6 h | | 26% | 51% | 23% | 36% |
| DOX 2.0 μg ml$^{-1}$ – 6 h | | 29% | 56% | 15% | 90% |
| DOX 2.0 μg ml$^{-1}$ + 5 μM TFP – 3 h | | 31% | 43% | 26% | 9.5% |
| DOX 2.0 μg ml$^{-1}$ + 5 μM TFP – 6 h | | 0% | 17% | 83% | 2.0% |

[a]% Fluorescent cells/fluorescence peak channel and cell cycle phase distribution are from a representative experiment; the standard deviations in replicate experiments were <15% of the means. [b]Mean value from triplicate plates in duplicate experiments; the standard deviations were <15% of the means. Survival is based on colony counts.

treated P388/DOX cells in soft-agar. At concentrations of 0.25 μg ml$^{-1}$ DOX and 0.5 to 1.0 μg ml$^{-1}$ DOX, the survival of P388/DOX cells treated with TFP was 15% and 0.7% respectively. Although, significant perturbations in cell cycle traverse of P388/DOX cells treated for 3 h and 6 h, were apparent only at 2.0 μg ml$^{-1}$ DOX + 5 μM TFP, cell kill in the presence of TFP was greater with increasing length of drug exposure both at 0.5 μg ml$^{-1}$ and 2.0 μg ml$^{-1}$ DOX.

The results on the effect of DAU or DAU + 5 μM TFP on cellular fluorescence, cell cycle traverse and cytotoxicity were similar to those described earlier with DOX. Additionally, in agreement with our earlier observations, on a molar basis, DOX was ~2-fold less potent than DAU in the presence of TFP (Ganapathi *et al.*, 1984*a*).

DOX-resistant cells are cross-resistant to DAU as well as lipophilic semisynthetic derivatives of DOX and DAU which differ in their cellular pharmacokinetics and binding properties to DNA (Ganapathi *et al.*, 1985). The results from the present study demonstrate, that in P388/DOX cells, at comparable cellular DAU or DOX levels, accumulation of cells in $G_2$ phase of the cell cycle and a reduction in colony formation due to enhanced cytotoxicity occurs only in the presence of TFP. In an earlier study using FCM to analyze the effects of TFP on DOX levels in P388/DOX cells treated for 2 h, it was observed that cell to cell heterogeneity in DOX fluorescence was apparent only in the absence of TFP. Based on these results, an enhanced cytotoxic effect with DOX in the presence of TFP was suggested to be due to a reduction in the heterogeneity of DOX accumulation (Ganapathi *et al.*, 1984*b*). Data from this study with P388/DOX cells treated for 24 h without TFP, indicates that cell to cell heterogeneity in DAU and DOX fluorescence is apparent only at the lower concentrations of 0.1 μg ml$^{-1}$ DAU and 0.25 μg ml$^{-1}$ DOX. In contrast, following treatment with TFP, both an absence of cell to cell heterogeneity in anthracycline accumulation and ~2-fold enhancement in DAU

**Figure 1** Representative histograms of data from **Table I** demonstrating the effect of the calmodulin inhibitor trifluoperazine on cellular fluorescence and cell cycle traverse of doxorubicin-resistant P388 mouse leukaemia cells treated with $0.25\,\mu g\,ml^{-1}$ daunorubicin (a, b, e and f) and $0.5\,\mu g\,ml^{-1}$ doxorubicin (c, d, g and h).

and DOX fluorescence was observed. Based on data in Figure 1, it can be argued that enhanced cytotoxicity with DAU and DOX in the presence of TFP is due to higher cellular anthracycline accumulation. However, it is apparent from the results in Table I, that at comparable cellular levels and cell to cell distribution of DAU and DOX fluorescence in P388/DOX cells, treated with two-fold higher concentrations of DAU or DOX in the absence of TFP versus half the concentration of DAU or DOX in the presence of TFP (e.g. $0.25\,\mu g\,ml^{-1}$ DAU *versus* $0.1\,\mu g\,ml^{-1}$ DAU + $5\,\mu M$ TFP; $0.5\,\mu g\,ml^{-1}$ DAU *versus* $0.25\,\mu g\,ml^{-1}$ DAU + $5\,\mu M$ TFP; $0.5\,\mu g\,ml^{-1}$ DOX *versus* $0.25\,\mu g\,ml^{-1}$ DOX + $5\,\mu M$ TFP; and $1.0\,\mu g\,ml^{-1}$ DOX *versus* $0.5\,\mu g\,ml^{-1}$ DOX + $5\,\mu M$ TFP), cell kill occurs only with TFP treatment. Thus, cellular levels *per se* do not appear to be a primary determinant of cytotoxicity or resistance to DAU and DOX in P388/DOX cells. Thin layer chromatographic analysis of extracts from P388/DOX cells treated with DAU and DOX, revealed little if any breakdown of drug, suggesting that resistance is probably not due to metabolism of drug to an inactive species (unpublished observations). Studies with DOX have demonstrated that an accumulation of cells in the $G_2$ phase of the cell cycle is related to a cytotoxic effect (Krishan & Frei, 1976). Since the P388/DOX cells were treated continuously with DAU and DOX for 24 h, which is ~2 cell cycle times, it appears that in the absence of TFP, the ability of the cells to overcome the $G_2$ block may be a determinant of resistance. Induction of cell cycle traverse perturbations in P388/DOX cells treated with DAU and DOX in the presence of TFP, is unrelated to the antiproliferative effects of the calmodulin inhibitor, since no cytotoxicity or alterations in cell cycle phase distribution were observed with TFP alone. It is therefore possible, that in the presence of TFP, the P388/DOX cells are unable to overcome the damage induced by DAU and DOX, which result in cell cycle traverse perturbations and cytotoxicity. This is supported by some of our recent studies, where we have found that a reversal of DOX induced $G_2$ block which parallels reduced cytotoxicity, does not occur in the presence of 5 $\mu$M TFP (unpublished observations). Resistance to DOX appears to be multifactorial (Ganapathi *et al.*, 1984*a*; Kessel & Wilberding, 1985) since treatment with TFP does not restore DOX-sensitivity comparable to the parent-sensitive line. The results from this study demonstrating a lack of relationship between cellular DOX and DAU levels and cytotoxicity are also supported by the recent study of Chang and Gregory (1985), wherein inherent resistance to DOX in pancreatic adenocarcinoma was found to be unrelated to cellular DOX levels. In summary, this study suggests, that the expression of resistance to the cytotoxic effects of DAU and DOX in DOX-resistant P388 mouse leukaemia cells is unrelated to gross cellular drug levels, and that treatment with TFP (which may alter intracellular compartmentalization of DAU and DOX) results in the induction and expression of damage caused by DAU and DOX. Although membrane alterations have been implicated to mediate DOX-resistance by altering drug permeation (Kartner *et al.*, 1983), it appears that a delineation of the intracellular mechanism of action of DAU and DOX may greatly aid our understanding of the phenomenon of resistance to these clinically important DNA-binding anthracyclines.

Supported by USPHS grant RO1 CA 35531 and CA 33505 awarded by the National Cancer Institute, Department of Health and Human Services. The authors gratefully acknowledge Nijole Mazelis for her excellent secretarial assistance and Joseph Kanasz of the Art-Medical Illustrations and Photography Department for skillful preparation of the art-work.

### References

CHAFOULEAS, J.G., BOLTON, W.E., HIDAKA, H., BOYD, A.E. III & MEANS, A.R. (1982). Calmodulin and the cell cycle: involvement in regulation of cell-cycle progression. *Cell*, **28**, 41.

CHANG, B.K. & GREGORY, J.A. (1985). Comparison of the cellular pharmacology of doxorubicin in resistant and sensitive models of pancreatic cancer. *Cancer Chemother. Pharmacol.*, **14**, 132.

DANO, K. (1976). Experimentally developed cellular resistance to daunomycin. *Acta Pathol. Microbiol. Scand.* (*A*)., **256** (Suppl.), 11.

FREI, E. III (1982). Models and the clinical dilemma. In *Design of Models for Testing Cancer Therapeutic Agents*, Fidler, I.J. & White, R.J. (eds) p. 248. Van Nostrand Reinhold Co.: New York.

GANAPATHI, R. & GRABOWSKI, D. (1983). Enhancement of sensitivity to Adriamycin in resistant P388 leukemia by the calmodulin inhibitor trifluoperazine. *Cancer Res.*, **43**, 3696.

GANAPATHI, R., GRABOWSKI, D., ROUSE; W. & RIEGLER, R. (1984*a*). Differential effect of the calmodulin inhibitor trifluoperazine on cellular accumulation, retention, and cytotoxicity of anthracyclines in doxorubicin (Adriamycin)-resistant P388 mouse leukemia cells. *Cancer Res.*, **44**, 5066.

GANAPATHI, R., GRABOWSKI, D., TURINIC, R. & VALENZUELA, R. (1984*b*). Correlation between potency of calmodulin inhibitors and effects on cellular levels and cytotoxic activity of doxorubicin (Adriamycin) in resistant P388 mouse leukemia cells. *Eur. J. Cancer Clin. Oncol.*, **20**, 799.

GANAPATHI, R., GRABOWSKI, D., SCHMIDT, H., SESHADRI, R. & ISRAEL, M. (1985*a*). Calmodulin inhibitor trifluoperazine selectively enhances cytotoxic effects of strong vs weak DNA binding antitumor drugs in doxorubicin-resistant P388 mouse leukemia cells. *Biochem. Biophys. Res. Commun.*, **131**, 912.

GANAPATHI, R., GULICK, P., MILLER, R. & 5 others. (1985*b*). Analysis of heterogeneity in daunorubicin uptake by human leukemia cells using laser flow cytometry. *Investigational New Drugs*, **3**, 273.

INABA, M. & JOHNSON, R.K. (1978). Uptake and retention of Adriamycin and daunorubicin by sensitive and anthracycline-resistant sublines of P388 leukemia. *Biochem. Pharmacol.*, **27**, 2123.

INABA, M., KOBAYASHI, H., SAKURAI, Y. & JOHNSON, R.K. (1979). Active efflux of daunorubicin and Adriamycin in sensitive and resistant sublines of P388 leukemia. *Cancer Res.*, **39**, 2200.

KARTNER, N., SHALES, M., RIORDAN, J.R. & LING, V. (1983). Daunorubicin-resistant Chinese hamster ovary cells expressing multidrug resistance and a cell-surface P-glycoprotein. *Cancer Res.*, **43**, 4413.

KESSEL, D. & WILBERDING, C. (1985). Anthracycline resistance in P388 murine leukemia and its circumvention by calcium antagonists. *Cancer Res.*, **45**, 1687.

KRISHAN, A. (1975). Rapid flow cytofluorometric analysis of mammalian cell cycle by propidium iodide staining. *J. Cell Biol.*, **66**, 188.

KRISHAN, A. & FREI, E. III (1976). Effect of adriamycin on the cell cycle traverse and kinetics of cultured human lymphoblasts. *Cancer Res.*, **36**, 143.

MUGGIA, F.M. & ROSENCWEIG, M. (1983). The anthracycline antibiotics: New directions in drug development and cancer treatment. In *Cancer Chemotherapy 1*, Muggia (ed) p. 123. Martinus Nijhoff Publishers: Boston.

RIEHM, H. & BIEDLER, J.L. (1972). Potentiation of drug effect by Tween 80 in Chinese hamster cells resistant to actinomycin-D and daunomycin. *Cancer Res.*, **32**, 1195.

SKOVSGAARD, T. (1978). Mechanisms of resistance to daunorubicin in Ehrlich ascites tumor cells. *Cancer Res.*, **38**, 1785.

SKOVSGAARD, T. (1980). Circumvention of resistance to daunorubicin by N-acetyldaunorubicin in Ehrlich ascites tumor. *Cancer Res.*, **40**, 1077.

TSURUO, T., IIDA, H., TSUKAGOSHI, S. & SAKURAI, Y. (1982). Increased accumulation of vincristine and Adriamycin in drug-resistant P388 tumor cells following incubation with calcium antagonists and calmodulin inhibitors. *Cancer Res.*, **42**, 4730.

WHEELER, C., RADER, R. & KESSEL, D. (1982). Membrane alterations associated with progressive Adriamycin resistance. *Biochem. Pharmacol.*, **31**, 2691.

YEN, A. & FAIRCHILD, D.G. (1982). T cell control of B cell proliferation uncoupled from differentiation. *Cell Immunol.*, **74**, 269.